# Altered phosphorylation, electrophysiology, and behavior on attenuation of PDE4B action in hippocampus

**DOI:** 10.1186/s12868-017-0396-6

**Published:** 2017-12-02

**Authors:** Susan L. Campbell, Thomas van Groen, Inga Kadish, Lisa High Mitchell Smoot, Graeme B. Bolger

**Affiliations:** 1 Department of Neurobiology, University of Alabama at Birmingham, Birmingham, AL 35294 USA; 2 Department of Cell, Developmental and Integrative Biology, University of Alabama at Birmingham, Birmingham, AL 35294 USA; 3 Department of Pharmacology, University of Alabama at Birmingham, Birmingham, AL 35294 USA; 4 Department of Medicine, University of Alabama at Birmingham, NP 2501, 1720 2nd Ave S, Birmingham, AL 35294-3300 USA; 5 Present Address: Center for Glial Biology in Health, Disease, and Cancer, Virginia Tech Carilion Research Institute, 2 Riverside Circle, Roanoke, VA 24016 USA

**Keywords:** Learning, Memory, Depression, PKA, CREB, DISC1, PDE4, PDE4B1

## Abstract

**Background:**

PDE4 cyclic nucleotide phosphodiesterases regulate 3′, 5′ cAMP abundance in the CNS and thereby regulate PKA activity and phosphorylation of CREB, which has been implicated in learning and memory, depression and other functions. The PDE4 isoform PDE4B1 also interacts with the DISC1 protein, implicated in neural development and behavioral disorders. The cellular functions of PDE4B1 have been investigated extensively, but its function(s) in the intact organism remained unexplored.

**Results:**

To specifically disrupt PDE4B1, we developed mice that express a PDE4B1-D564A transgene in the hippocampus and forebrain. The transgenic mice showed enhanced phosphorylation of CREB and ERK1/2 in hippocampus. Hippocampal neurogenesis was increased in the transgenic mice. Hippocampal electrophysiological studies showed increased baseline synaptic transmission and enhanced LTP in male transgenic mice. Behaviorally, male transgenic mice showed increased activity in prolonged open field testing, but neither male nor female transgenic mice showed detectable anxiety-like behavior or antidepressant effects in the elevated plus-maze, tail-suspension or forced-swim tests. Neither sex showed any significant differences in associative fear conditioning or showed any demonstrable abnormalities in pre-pulse inhibition.

**Conclusions:**

These data support the use of an isoform-selective approach to the study of PDE4B1 function in the CNS and suggest a probable role of PDE4B1 in synaptic plasticity and behavior. They also provide additional rationale and a refined approach to the development of small-molecule PDE4B1-selective inhibitors, which have potential functions in disorders of cognition, memory, mood and affect.

## Background

 Selective pharmacologic inhibition of the PDE4, 3′, 5′-cAMP-specific phosphodiesterases has been shown to produce antidepressant and memory-enhancing properties in humans [1–4] and rodents [5–12]. PDE4-selective inhibitors also have anti-inflammatory, immunomodulatory and smooth-muscle relaxant properties (see [13] for a review). Currently, three PDE4-selective inhibitors, roflumilast, apremilast and crisaborole, have been developed for clinical use, in respiratory and inflammatory disorders [14–16], and additional PDE4 inhibitors have been developed and tested for a variety of indications, including depression, schizophrenia, and disorders of learning and memory [17, 18].

By increasing intracellular levels of cAMP, PDE4-selective inhibitors activate cAMP-dependent protein kinase (PKA) and thereby increase its activity at numerous substrates, most importantly at the cyclic nucleotide response element binding protein (CREB), implicated in numerous functions, notably learning and memory (see [19–21] for reviews) and depression [22]. All PDE4-selective inhibitors act at the catalytic sites of the PDE4 enzymes [23–25] and therefore act, at least in part, as competitive inhibitors of cAMP hydrolysis. There are over 20 PDE4 isoforms, which are encoded by 4 genes in mammals, with additional diversity being produced by alternative mRNA splicing and the use of alternative promoters for each isoform [26, 27]. The catalytic sites of these isoforms are extremely similar, which has greatly complicated the development of inhibitors selective for any individual isoform [24]. However, the mRNA, and corresponding protein, for each isoform has a distinct pattern of expression in tissues, with significant regional differences in expression in the CNS, suggesting that each has a distinct tissue or organismal function [28–37].

To determine the functional status of specific PDE4 isoforms in the CNS, and thereby aid the targeting of drug development to isoforms that are functionally significant, we have adopted an isoform-selective approach. We and our collaborators first utilized this approach to study the PDE4D5 isoform in cell-based assays [38–40]. For this purpose, we developed a mutant in a specific metal-binding site in the catalytic region of the PDE4 protein that dramatically reduced its catalytic activity [38–41]. This mutation is in a single amino acid (D562A in PDE4D5, corresponding to D564A in PDE4B1, and conserved in all PDE4 isoforms) that was identified by X-ray crystallographic analysis as being essential for metal-ion binding and thus catalysis [42]. When over-expressed in cells, this mutant does not detectably change total PDE4 enzymatic activity in the cells where it is expressed, but is designed to produce an equilibrium displacement of the corresponding endogenous PDE4 isoform from its protein partner(s), or affect the ability of the PDE4 isoform to homodimerize [43–47], and therefore disrupt its cellular function in a dominant-negative fashion [38–41]. This approach is complementary to other genetic approaches, such as gene knockouts [48–53] and lentiviral siRNA [54–56], that have been used successfully to probe PDE4 functions in the CNS, but is potentially more isoform-selective.

**Fig. 1:**
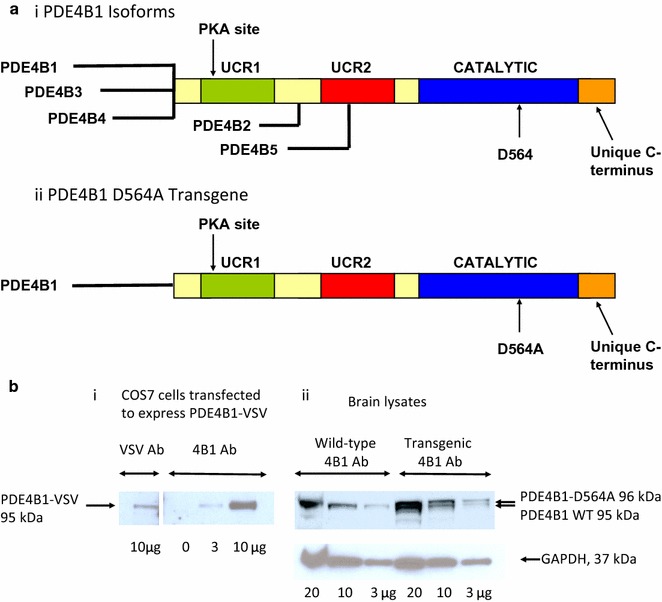
**a** (i) Schematic of PDE4B isoforms. The long isoforms PDE4B1, PDE4B3, and PDE4B4 contain the catalytic domain, UCR1 and UCR2, plus isoform-specific unique regions at their amino-termini. The short isoform PDE4B2 contains the catalytic domain and UCR2, while the super-short PDE4B5 isoform contains the catalytic region and a portion of UCR2. UCR1 and UCR2 mediate dimerization of the long PDE4B isoforms. Also shown are the carboxyl-terminal region common to all PDE4B isoforms, the PKA site located within UCR1, and the location of D564, the amino acid mutated in this study. (ii) Structure of PDE4B1 with the D564A mutation that is expressed in the transgenic mice. **b** Expression of the PDE4B1-D564A transgene. (i) Characterization of the PDE4B1 antibody. Extracts from COS7 cells transfected to express PDE4B1-VSV were immunoblotted with an antibody against VSV or against PDE4B1; both antibodies detected a protein of identical mobility, of 95 kDa. (ii) Immunoblotting of brain lysates from wild-type mice with the PDE4B1 antibody identified endogenous PDE4B1 (95 kDa). Lysates from PDE4B1-D564A transgenic mice also expressed a band of slightly slower mobility, representing the protein encoded by the PDE4B1-D564A transgene (96 kDa), in addition to endogenous PDE4B1 (95 kDa). Immunoblotting with GAPDH was used a loading control. The amount of extract protein loaded per lane is given at the bottom of each lane

We are especially interested in the PDE4B1 isoform, 1 of 5 isoforms encoded by the mouse *Pde4b* gene and which is highly conserved among mammals (Fig. 1a, Refs. [26, 57]). Each PDE4B isoform has a different pattern of expression in the CNS [27–29, 32, 33, 35, 37, 57–60], suggesting that each mediates a specific, non-overlapping function; however, the precise neurobiological functions of each of these isoforms will require additional investigation. *In*-*situ* studies have detected PDE4B1 mRNA expression in mouse hippocampal CA2 and CA3 regions, parietal and piriform cortex, and the cerebellar granular layer, among other brain regions [33], suggesting a potential role in a variety of CNS functions. PDE4B1 selectively interacts with several proteins, most notably DISC1 (Refs. [61–64]; see Ref. [65] for a review), and DISC1 appears to have a higher avidity for PDE4B1 than for any other PDE4 isoform [61, 62]. DISC1 is implicated in neurogenesis [65–69] and mutations in DISC1 have been shown to produce a schizophrenia-like phenotype in both mice [64, 70] and humans [71]. We were particularly interested in whether the PDE4B1-D564A mutant, when expressed as a transgene in the brain, might produce a DISC1-like phenotype, or show memory-enhancing or antidepressant effects.

## Results

### The PDE4B1-D564A mutant as a probe for PDE4B1 action in the CNS

**Fig. 2:**
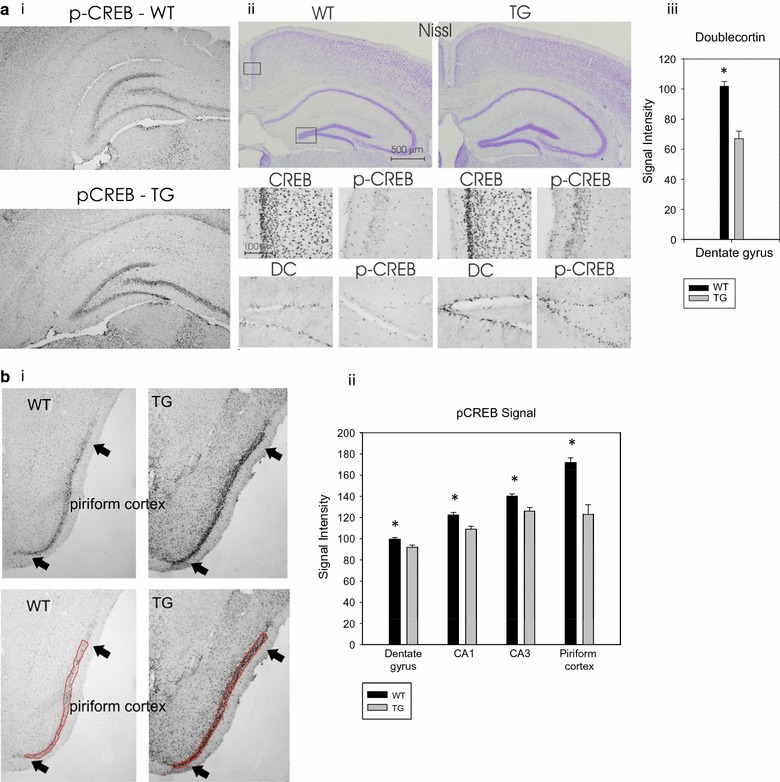
Increased pCREB, pERK and neurogenesis in PDE4B1-D564A transgenic mice. All data in the bar graphs was obtained using ImageJ, where an increased signal is indicated by a *lower* intensity. All data are mean ± SE. Asterisks (*) show data from the transgenic mice that is statistically different from that obtained from wild-type mice. **a** The PDE4B1-D564A transgene increases hippocampal pCREB and neurogenesis. (i) Immunodetection of pCREB in wild-type (WT) and transgenic (TG) mice in hippocampus. (ii) Select areas within the hippocampus. Top pair of panels: Nissl staining of hippocampus shows no major change between wild-type (WT) and transgene (TG). Small boxes indicate regions of CA1 and dentate gyrus shown at higher magnification in the middle and lower panels. Middle 4 panels: CA1 region: Levels of pCREB are increased in the transgenic mice, while total CREB is not detectably different. Lower 4 panels: Dentate gyrus: Doublecortin (DC)–labeled cells and pCREB levels are increased in the transgenic mice. (iii) Quantitation of DC-labeled cells in dentate gyrus, as determined by ImageJ. **b** The PDE4B1-D564A transgene increases pCREB in piriform cortex. (i) Top pair: Representative images of piriform cortex. Arrows indicate the region of most intense immunoreactivity. Bottom pair: Regions used for quantitation of pCREB expression are identified by red lines. (ii) Quantitation of pCREB immunoreactivity in 4 brain regions (**a**, **b**), as determined by ImageJ

We generated transgenic mice expressing the PDE4B1-D564A mutant (Fig. 1a, ii) under the control of the α-calmodulin kinase II (αCaMKII) promoter [72–74]. The founders were bred to wild-type C57BL/6 J mice and we determined that the PDE4B1-D564A transgene was inherited at the expected Mendelian frequency, demonstrating that the transgene did not affect fetal viability or otherwise show evidence of toxicity. PDE4B1-D564A transgenic mice had a gross phenotype that was indistinguishable from their wild-type littermates and from commercially-available C57BL/6 J mice. They also grew at a normal rate and reached a normal adult size. The protein encoded by the transgene, which had a VSV epitope [75] at its carboxyl-terminus and a charge difference because of its mutation, both of which would decrease its mobility under LDS-PAGE, was detectable as a band of slightly slower mobility on LDS-PAGE and immunoblotting (Fig. 1b). Microscopic morphology of the hippocampus in transgenic and wild-type mice was indistinguishable (Fig. 2a).

**Fig. 3:**
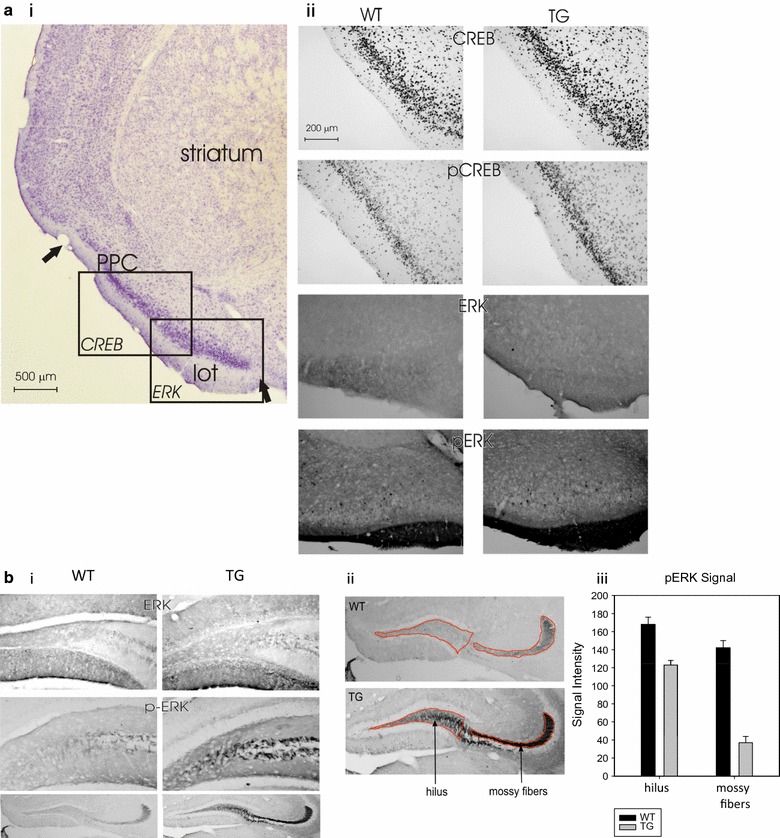
Increased pCREB, pERK and neurogenesis in PDE4B1-D564A transgenic mice. All data in the bar graphs was obtained using ImageJ, where an increased signal is indicated by a *lower* intensity. All data are mean ± SE. Asterisks (*) show data from the transgenic mice that is statistically different from that obtained from wild-type mice. **a** The PDE4B1-D564A transgene increases pCREB and pERK1/2 in prepiriform cortex and lateral olfactory tract. (i) Nissl staining. Boxes indicate regions shown in (ii); PPC: Prepiriform cortex; Lot: lateral olfactory tract. The arrow indicates the border of the piriform cortex. (ii) pCREB and pERK1/2 expression in PPC and Lot. Upper 4 panels: Expression of CREB and pCREB in prepiriform cortex in WT and TG mice, respectively. Lower 4 panels: Expression of ERK1/2 and pERK1/2 in the lateral olfactory tract of WT and TG mice, respectively. Increased pERK1/2 immunoreactivity was evident as a larger and more intense region of staining. **b** The PDE4B1-D564A transgene increases pERK1/2 in hippocampus. (i) Top pair: total ERK1/2 expression is not detectably different; ERK1/2 expression was highest near the surface of the gyrus. Second pair: pERK1/2 expression is increased in transgenic (TG), as opposed to wild-type (WT) mice in the mossy fibers projecting from the dentate gyrus. Third pair, wider field: pERK1/2 expression is increased in transgenic (TG), as opposed to wild-type (WT) mice in the mossy fibers projecting from the dentate gyrus. (ii) Enlarged views of pERK1/2 expression in dentate gyrus, with areas used for quantitation of pERK1/2 expression outlined in red. The hilus and mossy fibers are shown. (iii) Quantitation of pERK1/2 immunoreactivity in hilus and mossy fibers, as determined by ImageJ

### Increased CREB phosphorylation in PDE4B1-D564A transgenic mice

As phosphorylation of CREB (pCREB) is a sensitive indicator of changes in the activity of cAMP signaling pathways, we used phospho-specific antibodies to compare the expression of CREB and pCREB in PDE4B1-D564A transgenic and wild-type mice. These studies demonstrated increased levels of pCREB in PDE4B1-D564A transgenic mice, localized to several areas typical for the action of the αCaMKII promoter, notably area CA1 and the dentate gyrus of the hippocampus (Fig. 2a), the piriform cortex (Fig. 2b), the prepiriform cortex (Fig. 3a), and many nuclei of the hypothalamus (not illustrated). Total CREB expression was unchanged. Quantitation of pCREB immunoreactivity using ImageJ showed increased levels of pCREB in the dentate gyrus, area CA, area CA3 and piriform cortex in the transgenic mice, compared to wild-type (Fig. 2b, ii; increased expression of pCREB is indicated by a *lower* intensity by ImageJ, mean ± SE, all P < 0.01 by 2-way *t* test, n = 4). Indistinguishable changes were seen in mice of both sexes.

PDE4B1-D564A transgenic mice also demonstrated increased levels of pERK1/2 in the lateral olfactory tract (Fig. 3a) and 2 areas in the hippocampus (the hilus and the mossy fibers projecting from the dentate gyrus; Fig. 3b). Quantitation of hippocampal pERK1/2 immunoreactivity using ImageJ showed increased levels of pERK1/2 in the hilus and mossy fibers of transgenic mice, compared to wild-type (Fig. 3b, iii; increased expression of pERK1/2 is indicated by a *lower* intensity by ImageJ; all mean ± SE; n = 4; the results are typical of at least 4 mice and in animals of both sexes). In contrast, there was no change in ERK1/2 immunoreactivity in any area of the brain between PDE4B1-D564A transgenic and wild-type mice. As ERK1/2, like PKA, is a major regulator of CREB phosphorylation, these data are compatible with “cross-talk” between PKA and ERK signaling in regulation of CREB function in PDE4B1-D564A transgenic mice.

### Effect of the PDE4B1-D564A transgene on hippocampal neurogenesis

Enhanced hippocampal neurogenesis has been shown to be essential for most forms of learning, most notably fear conditioning [68, 76, 77], as well as for the action of several antidepressant drugs [54, 75, 78, 79]. Therefore, we used immunohistochemistry for doublecortin, a cellular proliferation marker, to assess neurogenesis. Doublecortin expression is an indication of newly-born neurons; however, on occasion, doublecortin expression can be seen in areas of the brain that lack neurogenesis. We detected increased doublecortin immunoreactivity in dentate gyrus and the rostral migratory stream in PDE4B1-D564A transgenic mice, as compared to wild-type mice (Fig. 2a), compatible with increased neurogenesis in these areas. Quantitation of doublecortin immunoreactivity using ImageJ showed an increased number of doublecortin-labeled cells in the dentate gyrus of transgenic mice, compared to wild-type (Fig. 2a, iii; an increased number of doublecortin-labeled cells is indicated by a *lower* intensity by ImageJ; all mean ± SE; n = 4; the results are typical of at least 4 mice and in animals of both sexes).

### The PDE4B1-D564A transgene had no detectable effect on basic neurological functions

We used the SHIRPA protocol [80, 81] to assess basic aspects of PDE4B1-D564A transgenic mice, including measurements of physical characteristics, general behavior, sensorimotor reflexes, and motor responses. The tests included measures of grip strength, the wire suspension test, and the Rotarod test. This testing was done with the objective of ensuring that the mice could see and hear normally and that they suffered from no motor deficits that would confound the results of any assays of learning and memory. PDE4B1-D564A transgenic mice were indistinguishable from their wild-type littermates in these assays.

### Effects of the PDE4B1-D564A transgene on behavior in a novel, open field

**Fig. 4:**
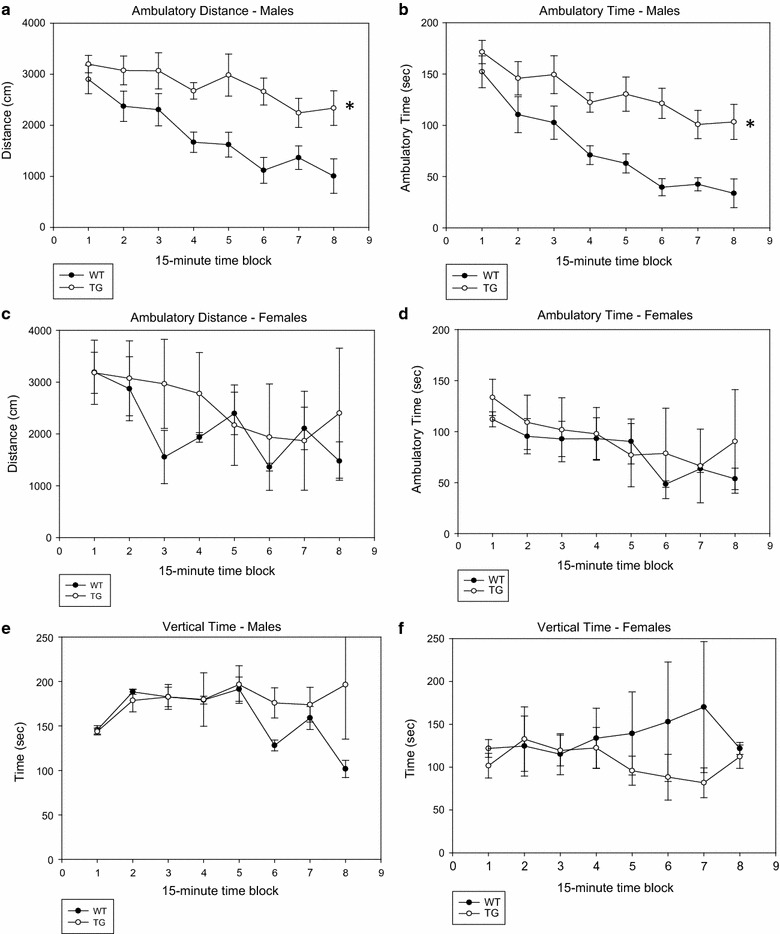
PDE4B1-D564A transgenic male mice show increased activity in an open field. Testing was performed for 2 h and activity analyzed for each 15-min block. Data are mean ± SE; asterisks (*) show data from the transgenic mice that is statistically different from that obtained from wild-type mice. **a** Ambulatory distance, males. **b** Ambulatory time, males. **c** Ambulatory distance, females. **d** Ambulatory time, females. **e** Vertical time, males. **f** Vertical time, females

**Fig. 5:**
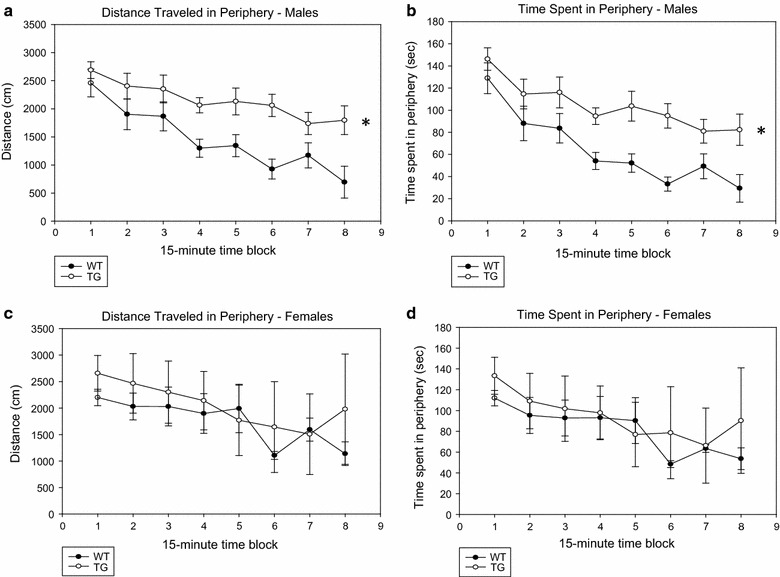
PDE4B1-D564A transgenic male mice show increased activity in an open field. Testing was performed for 2 h and activity analyzed for each 15-min block. Data are mean ± SE; asterisks (*) show data from the transgenic mice that is statistically different from that obtained from wild-type mice. **a** Distance traveled in periphery, males. **b** Time spent in periphery, males. **c** Distance traveled in periphery, females. **d** Time spent in periphery, females

In an open field, no significant difference was noted between PDE4B1-D564A transgenic and wild-type mice after only 10 min of testing. However, when activity was observed for 2 h, significant differences were noted in males (Figs. 4, 5). PDE4B1-D564A transgenic males, compared to wild-type males, had increased total activity (ambulatory distance, Fig. 4a, TG vs. WT, P = 0.000262, F = 45.75; for all male comparisons, n = 6 WT, n = 7 TG), and ambulatory time (Fig. 4b, P = 0.000129, F = 57.4). This effect was not seen in females (Fig. 4c, d, total ambulatory distance, TG vs. WT, P = 0.17, F = 2.11; total ambulatory time, P = 0.25, F = 1.41; for all female comparisons, n = 4 WT, n = 4 TG). No detectable effect was seen on vertical time (data for males, Fig. 4e, TG vs. WT, P = 0.172, F = 2.30, and for females, Fig. 4f, TG vs. WT, P = 0.054, F = 5.37). The effect of the mutant in males appeared robust, in that PDE4B1-D564A transgenic males also showed increased activity in the periphery of the field (ambulatory distance in the periphery, Fig. 5a, TG vs. WT, P = 0.000402, F = 39.7, and ambulatory time spent in the periphery, Fig. 5b, P = 0.00152, F = 54.4). This effect also was not seen in females (Fig. 5c, d, ambulatory distance in periphery, TG vs. WT, P = 0.16, F = 2.22, and ambulatory time in periphery, P = 0.25, F = 1.42).

As is typical for this assay, activity declined over the 2-h time course of the experiment. However, greater differences between the 1st and 2nd hours were seen in the wild-type males, reflecting the increased activity level overall in the PDE4B1-D564A transgenic male mice, producing a lower rate of fall in their activity over the 2-h time course (first vs. second hour, TG vs. WT: total ambulatory distance, P = 0.036, F = 5.38; total ambulatory time, P = 0.017, F = 7.34; distance in periphery, P = 0.019, F = 7.04; time in periphery, P = 0.020, F = 6.84, n = 6 WT, n = 7 TG).

### Lack of an anxiogenic effect of the PDE4B1-D564A transgene

**Fig. 6:**
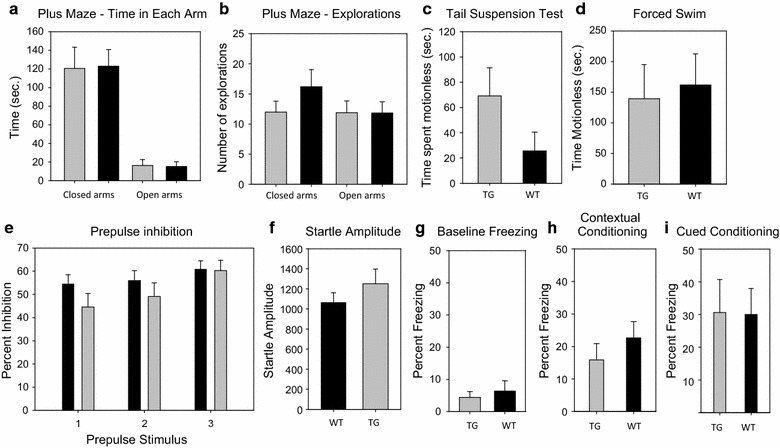
PDE4B1-D564A transgenic mice show no clear differences in tests of anxiety-like behavior, antidepressant-like action, or learning/memory. Black bars: Wild-type (WT). Grey bars: PDE4B1-D564A (TG). Data are mean ± SE. **a** Elevated plus maze, time in each arm. **b** Elevated plus maze, explorations of closed and open arms. **c** Tail suspension test, time spent motionless. **d** Forced swim test, time spent motionless. **e** Prepulse inhibition, percent inhibition with progressive stimulus (percent inhibition at pre-pulses of 4, 8 and 16 dB, column pairs 1, 2, and 3). **f** Prepulse inhibition, amplitude of startle response. **g** Fear conditioning, baseline freezing. **h** Fear conditioning, contextual conditioning. **i** Fear conditioning, cued conditioning

A potential anxiogenic effect of the PDE4B1-D564A transgene was assessed with the elevated plus maze. Both time spent in the open arms and exploration attempts were tested; the data shown are pooled for both sexes (Fig. 6a, b). We were unable to detect differences between PDE4B1-D564A transgenic and wild-type mice in this assay. Each genotype spent an indistinguishable amount of time in each arm (TG vs. WT, sexes pooled: P = 0.91 for closed arms, F = 0.013; P = 0.88 for open arms, F = 0.023; n = 10 WT, n = 10 TG), with no detectable difference by sex (females: P = 0.98 for closed arms, F = 0.009; P = 0.13 for open arms, F = 2.86; n = 5 WT, n = 5 TG; males: P = 0.14 for closed arms, F = 21.52; P = 0.022 for open arms, F = 791; n = 5 WT, n = 5 TG). Explorations into each arm were also indistinguishable (sexes pooled: P = 0.15 for closed arms, F = 2.28; P = 0.98 for open arms, F = 0.00046), with no detectable difference by sex (females: P = 0.094 for closed arms, F = 3.48; P = 0.34 for open arms, F = 0.99; males: P = 0.72 for closed arms, F = 0.21; P = 0.37 for open arms, F = 2.25).

### Lack of an antidepressant effect of the PDE4B1-D564A transgene

Some PDE4-selective inhibitors, such as rolipram, have antidepressant action in humans [1–4] and rodents [5–12]. We therefore tested the antidepressant action of the PDE4B1-D564A transgene in the tail-suspension and forced-swim tests [82–86]. The data shown were pooled for both sexes (Fig. 6c, d). No antidepressant effect of the PDE4B1-D564A transgene was apparent in the tail-suspension test (TG vs. WT, time spent absolutely motionless, sexes pooled: P = 0.086, F = 3.83, n = 9 WT, n = 10 TG; males: P = 0.09, F = 4.9, n = 5 WT, n = 5 TG; females: P = 0.60, F = 0.34, n = 4 WT, n = 5 TG). Given the P values that we obtained in these studies, which are approaching 0.1, it is possible that the transgene produced a weak effect that might have become statistically significant with a much larger sample size. We also saw no effect in the forced-swim test (time spent absolutely motionless, except for respirations or drifting, sexes pooled: P = 0.86, F = 0.477, n = 10 WT, n = 10 TG; males: P = 0.72, F = 0.209, n = 5 WT, n = 5 TG; females: P = 0.78, F = 0.44, n = 4 WT, n = 5 TG).

### Lack of effect of the PDE4B1-D564A transgene on acoustic startle and pre-pulse inhibition

The PDE4B1 and DISC1 proteins directly interact and PDE4B1 appears to influence the phosphorylation status of DISC1 [61–63, 87, 88]. Mutations of mouse DISC1 have a diverse behavioral phenotype that includes alterations in pre-pulse inhibition [36, 64, 70]. Therefore, we tested the effect of the PDE4B1-D564A transgene in this assay. Although PDE4B1-D564A transgenic and wild-type mice had similar responses to acoustic startle, we were unable to detect any difference in pre-pulse inhibition (Fig. 6e, f; percent inhibition at pre-pulses of 4, 8 and 16 dB above background, TG vs. WT, sexes pooled: P = 0.15, 0.35 and 0.91, respectively, F = 2.18, 0.92 and 0.014, respectively; for males: P = 0.13, 0.21 and 0.64, respectively, F = 2.72, 1.79, 0.23, respectively; for females: P = 0.86, 0.92, 0.60, respectively, F = 0.033, 0.011, and 0.30, respectively). Startle amplitude was very similar (sexes pooled: P = 0.29, F = 1.20; n = 10 WT, n = 12 TG; for males: P = 0.15, F = 2.41; n = 6 WT, n = 6 TG; for females: P = 0.63, F = 0.266, n = 4 WT, n = 6 TG).

### Lack of effect of the PDE4B1-D564A transgene on fear-associated conditioning

Given the enhanced CREB and ERK1/2 phosphorylation seen in PDE4B1-D564A transgenic mice, we wished to determine whether these mice demonstrated improved function in assays for learning and memory. Deficiencies in fear-associated conditioning are a hallmark of mice deficient in CREB [20, 21, 89–93]. We tested our mice in a typical fear-associated conditioning protocol and measured baseline freezing (Fig. 6g) and both context-dependent and cue-dependent conditioning (Fig. 6h, i). We could not detect differences between PDE4B1-D564A transgenic and wild-type mice in baseline freezing (percent freezing, TG vs. WT, sexes pooled: P = 0.62, F = 0.258, n = 12 WT, n = 11 TG; for males: P = 0.22, F = 1.63, n = 6 WT, n = 7 TG; for females: P = 0.48, F = 0.54, n = 6 WT, n = 4 TG). We also detected no difference in freezing after cued conditioning (percent freezing with stimulus, sexes pooled: P = 0.96, F = 0.002; n = 12 WT, n = 11 TG; for males: P = 0.77, F = 0.092, n = 6 WT, n = 7 TG; for females: P = 0.57, F = 0.35, n = 6 WT, N = 4 TG). We also detected no difference in freezing after contextual conditioning (percent freezing, sexes pooled: P = 0.35, F = 0.92; for males: P = 0.24, F = 1.54; n = 6 WT, n = 7 TG; for females: P = 0.99, F = 0.00001, n = 6 WT, n = 4 TG).

### PDE4B1-D564A transgenic mice demonstrated altered hippocampal synaptic transmission

**Fig. 7:**
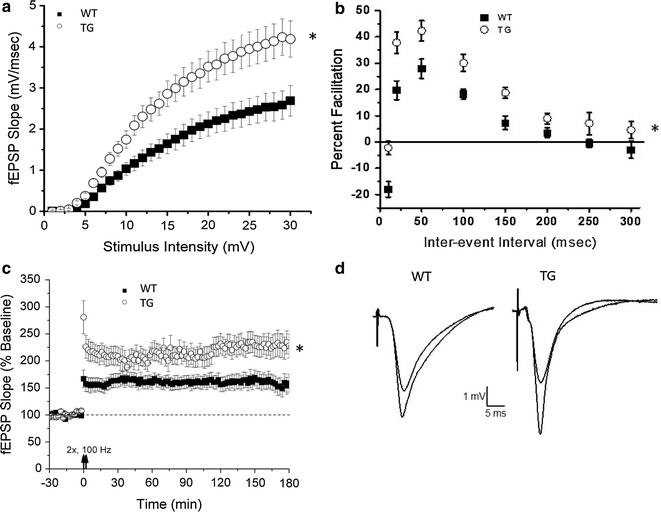
Synaptic transmission and plasticity are enhanced in male PDE4B1-D564A transgenic mice. Data are mean ± SE; asterisks (*) show data from the transgenic mice that is statistically different from that obtained from wild-type mice. **a** Baseline synaptic transmission shown with input–output curves for wild-type (WT) and PDE4B1-D564A (TG) male mice. **b** Paired-pulse facilitation in hippocampal slices. **c** Long-term potentiation (LTP) induced with a pair of 100-Hz tetani in area CA1. The arrows indicate the delivery of 2 trains of 100-Hz, 1-s stimulation. **d** Representative traces obtained 4 min before (black), and 2 h after LTP (gray), in wild-type (WT) and PDE4B1-D564A (TG) male mice

Mice with mutations that block or down-regulate CREB show alterations in hippocampal long-term potentiation (LTP), underlying learning and memory [20, 21, 89–94]. Hippocampal slice electrophysiology was therefore used to study male PDE4B1-D564A transgenic mice and wild-type controls. PDE4B1-D564A transgenic mice had elevated baseline synaptic transmission at Schaffer collateral synapses. Input–output functions generated by stimulation of area CA3 and field recordings of pyramidal neurons in area CA1 were enhanced in PDE4B1-D564A transgenic mice (Fig. 7a; F_(1,729)_ = 61, P < 0.00001). Paired-pulse facilitation (PPF) was also assessed. This is a form of short-term plasticity believed to result from residual calcium in the presynaptic cell that enhances neurotransmitter release. PPF in PDE4B1-D564A transgenic mice was significantly elevated over wild-type controls at all interstimulus intervals tested (Fig. 7b; F_(1,624)_ = 48, P < 0.0001). These results suggest that the PDE4B1-D564A transgene is capable of affecting normal hippocampal synaptic transmission and may be an integral component of the machinery underlying short-term plasticity. Given the role of PKA in the induction of LTP, we analyzed hippocampal LTP in PDE4B1-D564A transgenic mice. In these experiments, LTP was induced with two 1-sec, 100-Hz tetani separated by 20 s. We observed a significant difference between PDE4B1-D564A transgenic mice and wild-type littermate controls in post-tetanic potentiation (PTP) and LTP (Fig. 7c, d). At 2 h after LTP induction, the fEPSP slope was 131 ± 17% (WT) and 231 ± 19% (TG) of baseline, respectively; F_(1,113)_ = 6.9, p = 0.009. As members of Kandel’s group have shown, typically 3 tetani are required to induce PKA-mediated L-LTP [95–97]; therefore our conclusion that the changes in LTP that we see here are indeed due to PKA action must be interpreted with some caution. Also, the combination of enhanced input–output with increased LTP that we see here is unusual, as increased basal transmission is typically associated with reduced LTP due to a ceiling effect. We attribute the increased LTP to the strong signal that we see with two tetani. Overall, these data indicate that area CA1 hippocampal physiology, in terms of baseline synaptic transmission, PPF, and tetanus-induced long-term plasticity, can be modulated by PDE4B1.

## Discussion

In this study, we have generated transgenic mice that express a mutant (D564A) of an individual PDE4 isoform, specifically PDE4B1, preferentially in the hippocampus and forebrain, and tested the effect of the mutant on synaptic plasticity, phosphorylation, and behavior. Our use of an isoform-selective PDE4 mutant as a transgene is a novel approach to determining the functions of PDE4 isoforms in the CNS, and can be contrasted to previous studies of PDE4 function employing gene knockouts [48–53, 98] or lentiviral siRNA [54–56]. As a precedent for this approach, we and our collaborators have studied successfully, in cell-based assays, the corresponding mutation in a different PDE4 isoform, specifically PDE4D5 [38–40]. The use of our PDE4B1 mutant, expressed as a transgene, has the potential to be more isoform-selective than the PDE4B gene knockouts that have been studied to date: The mouse *Pde4b* and human *PDE4B* gene both encode 5 isoforms [26–28, 57–60], each with a distinct protein structure and pattern of expression in tissues. Therefore, *Pde4b*−/− mice have a phenotype that reflects the combined deficiency of all 5 PDE4B isoforms, which greatly complicates analysis of the effect(s) of any individual isoform, such as PDE4B1. For this reason, it is reasonably likely that our approach has greater specificity for PDE4B1 than gene knockouts or siRNA. Our generation of mutants as transgenes also follows a strategy used by other groups in the study of PKA and CREB in the CNS: e.g., transgenic mice expressing a dominant-negative PKA RIα subunit [97], or a dominant-negative CREB mutant [21, 90–92, 94, 99–101], all have defects in various aspects of learning and memory.

For this study, we expressed the PDE4B1-D564A transgene off the αCaMKII promoter [72–74]. This promoter is active preferentially in excitatory neurons of forebrain areas, including the hippocampus, amygdala, cortex and striatum [72, 73]. The transcription of mRNA driven by the αCaMKII promoter is silent until several days after birth [102], when many neural circuits are already formed, thereby possibly minimizing any adverse effects of the transgene during pre-natal development of the brain [74]. This promoter has been used widely in the development of both transgenic and knockout mice used in the study of learning and memory [72, 74, 84, 90–92, 97, 103, 104] and other neurobiological phenotypes [105]. It therefore seemed to be a reasonable choice for our studies. However, there are certainly differences in the regional expression in brain of αCaMKII and PDE4B1; therefore, we cannot guarantee that, in our mice, all of the biological functions of PDE4B1 are disrupted equally in all regions of the brain where it is normally expressed. It is also likely that our PDE4B1-D564A transgene is expressed in at least some brain areas lacking endogenous PDE4B1; however, since the only known function of PDE4B1-D564A is mediated through its action on endogenous PDE4B1 (see next paragraph for further details), the transgene is unlikely to have a detectable biological effect in those areas.

When over-expressed in cells, as is the case here, our PDE4B1-D564A mutant does not detectably change total PDE4 enzymatic activity in those cells, but is designed to produce an equilibrium displacement of endogenous PDE4B1 from its protein partner(s), or affect the ability of PDE4B1 to homodimerize [43–47], and therefore disrupt its cellular function. We cannot exclude completely some “off-target” effect of the PDE4B1-D564A protein on other PDE4 isoforms, especially other PDE4B isoforms. However, since PDE4B1 homodimerizes only with itself and also interacts with a different set of protein partners than do other PDE4B isoforms (including its preferential association with DISC1, as discussed below), we believe this to be unlikely. Therefore, expression of PDE4B1-D564A should lower PDE4B1 activity in specific sub-cellular compartments where PDE4B1 is active, increase local cAMP levels, and thereby activate PKA. Our immunohistochemistry data are consistent with this mechanism, as they show that phosphorylation of CREB, a major PKA substrate, is elevated in specific CNS regions where our transgene would be expected to be expressed, such as area CA1 of the hippocampus and the dentate gyrus. Although it is possible that a major phenotypic effect of the PDE4B1-D564A transgene is mediated by pCREB, it is certainly possible that some of its effect is mediated by other cAMP effectors, such as cyclic nucleotide-gated ion channels [106] or EPAC [107]. We note that total-tissue levels of cAMP need not change detectably with the PDE4B1-D564A transgene, as global cAMP levels are the cumulative effect of numerous elements in cAMP signaling, including other PDEs and the effects (possibly compensatory) of the other 19 PDE4 isoforms that have been discovered to date.

We chose to study PDE4B1 for several reasons. First, PDE4B1 mRNA is expressed in mouse hippocampal CA2 and CA3 regions, parietal and piriform cortex, and the cerebellar granular layer, among other brain regions [33], making it a reasonable candidate for attenuation by a transgene driven by the αCaMKII promoter. Secondly, PDE4B1 selectively interacts with several proteins, most notably DISC1 (Refs. [61–64]; see Ref. [65] for a review), and DISC1 appears to have a higher avidity for PDE4B1 than for any other PDE4 isoform [61, 62]. DISC1 is implicated in neurogenesis [65–69] and mutations in DISC1 have been shown to produce a schizophrenia-like phenotype in both mice [64, 70] and humans [71]. Therefore, study of PDE4B1 might provide insights into DISC1 function.

Our PDE4B1-D564A transgenic mice have a behavioral phenotype that affects only activity, given their phenotype in the open-field test (Figs. 4, 5). This phenotype, where activity is similar at the start of a 2-h test period but then diverges during the test period, is relatively non-specific but is also seen in mice with mutations in *Disc1* [64, 70]. However, we saw no effect of the PDE4B1-D564A transgene on pre-pulse inhibition, a schizophrenic-associated phenotype; in this regard, our mice differ substantially from *Disc1* mutant mice [70]. The PDE4B1-D564A transgene does not appear to have an anxiogenic effect, in view of PDE4B1-D564A transgenic mice having behavior similar to wild-type in the elevated plus maze (Fig. 6a, b). Our open-field data, which shows that our mice have a preference for the periphery of the test chamber, can be interpreted as an anxiogenic effect, but is more likely to reflect the overall increased activity seen in this test. Although rolipram and other PDE4-selective inhibitors have antidepressant activity, we saw no major antidepressant action of the PDE4B1-D564A transgene in the forced-swim or tail-suspension tests (Fig. 6c, d).

We also saw no effect of the PDE4B1-D564A transgene on associative fear conditioning. We tested fear conditioning as it is an amygdala-dependent memory test, with the contextual fear portion mediated, at least in part, by CREB action in the hippocampus, and because we detected increased pCREB in hippocampal areas CA1 and the dentate gyrus. Deficiencies in fear-associated conditioning are also a hallmark of mice deficient in CREB [20, 21, 89–93]. One possibility for the lack of augmented performance on fear conditioning in PDE4B1-D564A transgenic mice is that the baseline performance of the C57BL/6 J strain in this assay was already sufficiently high that any improvement might have been difficult to measure, especially when strong stimuli were used in conditioning, as was the case here. It is possible that effects of the PDE4B1-D564A transgene are most prominent in other neuronal pathways, such as the dentate gyrus (perforant pathway), which has been linked to several memory phenotypes [108, 109], including, but not necessarily limited to, classical fear conditioning. Increased plasticity in the perforant pathway could also explain the increased activity seen in our mice in open field testing, as it has been linked to the drive to explore [76, 110, 111]. It is also possible that a weak behavioral effect might have been missed because of sample size. In particular, a small sample size may have contributed to our inability to demonstrate a potential weak effect of the transgene in the tail suspension test (Fig. 6c, d). However, sample sizes were limited by resource and appropriate animal ethics issues.

*Pde4b*−*/*− mice have been studied by a number of groups, with disparate results [49, 51–53, 112]. Some studies of *Pde4b*−*/*− and *Pde4d*−*/*− mice have shown them to have behavioral characteristics that mimic the actions of antidepressants [48, 51, 52, 112]; for example, decreased immobility in tail-suspension and forced-swim tests. Some studies of *Pde4d*−*/*− mice have shown them to have augmented activity in tests of learning and memory [54], while studies of the identical genotype by other groups do not show this effect [50]. Study of all PDE4 knockouts have been complicated by non-CNS effects [113, 114], such as slow growth, small adult size and impaired fertility. In addition, assessment of the CNS phenotype of the knockouts has also been complicated by the fact that the knockouts appear to have an effect in numerous areas of the brain, most notably the striatum, in addition to the forebrain/hippocampus, which is the focus of the present study.

McGirr et al. [115] have reported recently the phenotype of mice with the *Pde4b* homozygous germline mutation Y358C. Like our D564A mutation, the Y358C mutation is present in the catalytic site of the enzyme (Fig. 1a) and thereby substantially abolishes its enzymatic activity. However, because their mutation is present in all isoforms encoded by the *Pde4b* gene, its phenotype is unlikely to be mediated by any single isoform. Additionally, it is expressed off the endogenous *Pde4b* promoter(s) and therefore is likely to have phenotypic effects outside the CNS. In contrast, our PDE4B1-D564A mutant is confined to a single isoform, PDE4B1, and is expressed off a transgene that is relatively specific for discrete regions of the CNS, as discussed above. The phenotype observed by McGirr et al. [115] is similar to that seen in our PDE4B1-D564A transgenic mice in that it displays increased activity in open field testing. Their mutant is also similar to PDE4B1-D564A transgenic mice in that it appears to have minimal effect on pre-pulse facilitation and on tests for an anti-depressant effect, such as the forced-swim test. Also similar to our PDE4B1-D564A transgenic mice, contextual fear learning at 24 h is intact in their mutant [115]. In contrast, our PDE4B1-D564A transgenic mice differ from those of McGirr, et al. in that we see no effect in assays for anxiety, such as the elevated plus maze. The differences between our findings and those of McGirr, et al., probably reflect isoform differences (PDE4B1 vs. all PDE4B isoforms), and/or the relative expression of the mutant (endogenous promoter(s) vs. transgene), although differences in genetic background, age at the time of study, or assay conditions could also be responsible.

Our data suggest strongly that PDE4B1 has an important role in the brain. Therefore, our results provide additional justification for the focusing on PDE4B1 as a target for drug development. However, CNS-mediated side effects common to PDE4-selective inhibitors, most notably emesis [24], may still be a major obstacle to development of PDE4B1-selective inhibitors for CNS disorders. Several PDE4B and PDE4D isoforms are expressed in the area postrema [35], critical in the mediation of emetogenic stimuli, suggesting that much of the pro-emetogenic effects of these agents are due to PDE4 inhibition in this area. Many of the emetogenic effects of PDE4 inhibitors appear to be mediated by PDE4D isoforms [116], but recent studies implicate PDE4B isoforms as well [12]. However, targeting PDE4 informs or conformers that are not present in the area postrema may still be therapeutically possible.

## Conclusions

We have demonstrated the value of an isoform-selective approach to investigating the function of an important PDE4 isoform, specifically PDE4B1. Our PDE4B1-D564A transgenic mice demonstrate increased phosphorylation of CREB and ERK1/2, consistent with activation of PKA, ERK1/2 and potentially other kinases, increased hippocampal neurogenesis, and alterations in hippocampal slice electrophysiology consistent with augmentation of hippocampal LTP. Behaviorally, we see increased activity in these mice, but no major changes in associative learning or other behavior. These data provide additional rationale for the development of small-molecule PDE4 inhibitors, which have potential functions in disorders of cognition, memory, mood and affect.

## Methods

### Generation of transgenic mice

We generated transgenic mice that expressed PDE4B1-D564A under the control of the α-calmodulin kinase II (αCaMKII) promoter. The PDE4B1-D564A mutant (Asp564Ala, GAC to GCC; GenBank AF202732, Ref. [57]) was cloned into pMM403 (Refs. [72, 73]; generously provided by Mark Mayford, Scripps Institute, USA), to provide a 5′ intron splice site and a 3′ intron and poly A sites. The plasmid construct containing the transgene was linearized at a unique *Sfi* I site and then injected into the pronuclei of C57BL/6 mice. All transgenic mice were generated on the C57BL/6 background, using a breeding colony maintained for that purpose at the Transgenic and Genetically-modified Mice Shared Facility at the University of Alabama at Birmingham (UAB) and subsequently backcrossed into purchased C57BL/6 J mice (The Jackson Laboratory, USA). The genotypes of the progeny mice were verified by PCR primers specific to the *Pde4b* gene in the transgene construct. No surgery was performed and all efforts were made to minimize suffering. Prior to the collection of tissue, animals were euthanized by CO_2_ inhalation. The experimenter was blind to the genotype of the mice when the testing was performed and data collected. All animal work was performed under protocols approved by the Institutional Animal Care and Use Committee (IACUC) of UAB and followed the NIH guide for the care and use of laboratory animals and other national regulations and policies. The IACAC specifically approved all animal protocols (Animal Project Number: 08010842) prior to the initiation of the study.

### Immunoblotting

Monkey COS7 cells were transfected with the plasmid pcDNAVSVR89 to express rat PDE4B1, or with vector pcDNA3 (Invitrogen, ThermoFisher, USA), using methods that we have described previously [57]. The COS7-expressed proteins were engineered to contain a VSV epitope at their amino-terminus. Extracts from COS7 cells were prepared using methods we have described previously [57]. Whole brain tissue from wild-type and transgenic mice was dissected and flash-frozen in liquid nitrogen. Extracts from brain were homogenized with a Polytron (Brinkmann, USA) in 50 mM Tris–HCl, pH 8.0, 150 mM NaCl, 0.1% SDS, 0.5% Na deoxycholate, and 1% IGEPAL CA-630 (all from Sigma, USA) plus Complete Protease Inhibitor (Roche Molecular Systems, USA) at room temperature for 10 s [117]. The extracts were analyzed by LDS-PAGE (Novex, Invitrogen, ThermoFisher, USA) and immunoblotted with either an antibody to VSV (mouse, clone P5D4, Sigma, Ref. [75]) or a PDE4B1 antibody (mouse, C173292, LS Bio, USA). Immunoblotting for GAPDH (rabbit, clone 14C10, Cell Signaling Technologies, USA) was used as a loading control. For comparing apparent molecular weights under denaturing conditions, samples were run in parallel lanes on the same gel and then transferred to a single filter. For immunoblotting with the PDE4B1 and GAPDH antibodies, the filter was then cut in half cross-wise and each half incubated with the appropriate antibody. Primary antibody incubations (1:2000 for PDE4B1, 1:5000 for GAPDH, and 1:5000 for VSV) were performed for 1 h in Tris-buffered saline (TBS) with 0.1% Tween-20, followed by 2 washes in the same buffer. Secondary antibody incubations (SC-5099, Santa Cruz, USA, 1:10,000) were performed in the same buffer, followed by 2 washes in the same buffer. Signal development was with ECL (Pierce, ThermoFisher, USA).

### Immunohistochemistry

Antibody staining in the CNS of transgenic and wild-type mice (2–3 mice in each experimental group, or as specified in Results) was performed as described previously [118]. Mice were anesthetized, decapitated and the brains were removed and stored in 4% paraformaldehyde overnight. Following cryoprotection in 30% sucrose, 6 series (1 in 6) of coronal sections were cut through the brain on a sliding, freezing microtome. The first, second, and third series were stained immunohistochemically as we have described [118]; the other 3 series were stored at − 20 °C in antifreeze for future analysis. One half of the 4th series was stained for DARPP-32 (rabbit, Cell Signaling, #2306). The other half was stained for doublecortin (rabbit, Cell Signaling, #4604). One half of the 5th series was stained for phospho-CREB (pCREB, rabbit, Cell Signaling, #9198), whereas the other half was stained for CREB (rabbit, Cell Signaling, #9197). One half of the 6th series was stained for phospho-ERK1/2 (pERK1/2, pT202/pY204, rabbit, Cell Signaling, #9101), whereas the other half was stained for ERK1/2 (rabbit, Cell Signaling, #9102). The series of sections were transferred to a solution containing the primary antibody (1:1000 dilution) in TBS with 0.5% Triton X-100 (TBS-T). Following incubation in this solution for 18 h on a shaker table at room temperature (20 °C) in the dark, the sections were rinsed 3 times in TBS-T and transferred to the solution containing the appropriate secondary antibody (goat anti-mouse*biotin or donkey anti-rabbit*biotin, Sigma). After 2 h, the sections were rinsed 3 times with TBS-T and transferred to a solution containing mouse ExtrAvidin^®^ (Sigma). Following rinsing, the sections were incubated for approximately 3 min with Ni-enhanced DAB [119, 120]. All stained sections were mounted on slides and coverslipped.

#### Immunochemistry controls and comparisons

All staining for pCREB and for pERK1/2 was performed together (i.e., on parallel slides) with staining for CREB and CalbindinD-28k (Cell Signaling, C26D12, #2173). Since in all cases we saw no change (WT vs. TG) in immunoreactivity for CREB or CalbindinD-28k, we used these slides as controls for staining for pCREB and/or pERK1/2.

#### Quantitation of immunochemistry data

The specificity of the pCREB and pERK1/2 antibodies employed here has been reported by a number of other groups, working on mouse brain tissues [121–124]. Quantitation of the signal produced with the pCREB antibody in mouse brain has been validated by several other groups [121, 122, 124, 125]. Stimulus-dependent increases in pCREB and/or pERK1/2, as detected by these antibodies, has also been reported by these groups [121–125]. Appropriate regions of hippocampus were selected (see Figs. 2, 3 for the specific areas) and grain intensity in each region was quantitated by ImageJ software (NIH, Bethesda, USA). Means ± standard errors were reported and comparisons made with a 2-way *t*-test (Excel, Microsoft, USA).

### Behavioral assays

Mice were tested at 8–12 weeks of age. The experimenter was blind to genotype in all behavioral experiments. Mice were housed in cages containing from 2 to 5 animals (except for 1 male mouse in cohort 2, below, which was housed singly), on a 12-h light, 12-h dark cycle. All behavioral experiments were performed in the light phase, except as specified below. All mice were tested first in a basic behavioral battery (i.e., SHIRPA testing, which included Rotarod), followed by at least a 5–7-day rest period. Open field testing, followed about 7 days later by fear conditioning and then acoustic startle, were performed on a single cohort of mice (Cohort 1; 2 generations backcrossed into C57BL/6 J; several mice were added to this cohort after the SHIRPA data were collected); many of these mice were then sacrificed for electrophysiological studies. For the elevated plus maze, tail suspension and forced swim tests (each performed about 1 week apart, in that order; one mouse was lost during a “rest” interval), a different cohort of mice was used (Cohort 2; 3 generations back-crossed into C57BL/6 J). Males and females were tested throughout and results were analyzed separately for each sex; if the results (WT vs. TG) were not statistically significant when sexes were separated in this fashion, the results for both sexes were pooled and statistical tests performed on this pooled sample set. Graphs were generated with SigmaPlot (Systat, Inc., USA).

#### Basic neurobiologic battery

Basic neurobiological observations were performed according to the SHIRPA protocol [80, 81]. It included measurements of physical characteristics, general behavior, sensorimotor reflexes, and motor responses (including grip strength, the wire suspension test, and the Rotarod test).

#### Open-field testing

This was performed as described previously [126–128], using a photobeam apparatus (Med Associates, St. Albans VT, USA). Each mouse was placed in the lower left corner of a clear arena (40 × 40 × 30 cm) in low-light conditions (12.5 lx); the sides of the arena were neutral and opaque. This open field was divided into 256 equally sized squares by 16 photoreceptor beams on each side of the arena. Locomotor activity was quantified with a Digiscan optical animal activity system (RXYZCM, Accuscan Instruments, Columbus OH, USA). Activity was measured by the number of photobeam interruptions in both the horizontal and vertical planes collected in 1-min intervals over a 120-min testing period, analyzed in 15-minute blocks. Additional activity data included the total distance traveled in the horizontal plane (in cm). We also measured activity and distance traveled in the periphery (i.e., outside the 16 most centrally located squares). Data are presented with means ± standard errors and statistical analysis was by a 2-way ANOVA, with genotype and sex the 2 between-subject factors.

#### Elevated plus maze

For the elevated plus maze, a standard test for measuring anxiety-like behavior [129], we used a 4-arm maze located about 100 cm from the floor (Med Associates). Each arm was 60 cm long; 2 were “open” and 2 had 20-cm high walls (“closed”) and testing was performed in low-light conditions (12.5 lx). Each trial was 5 min long and the mice were tracked with a continuous video tracking system (Noldus, The Netherlands). Anxiety-like behavior was observed by comparing the amount of time the mouse spent in the open arms (“unsafe”), compared to the closed arms (“safe”), and also by the number of entrances to each arm, as defined by the tracking system software. Normal mice spent some time in each arm, while anxious mice spent a greater proportion of their time in the closed arms. Data are presented with means ± standard deviations and statistical analysis was by a 2-way ANOVA, with genotype and sex the between-subject factors.

#### The tail-suspension test

This test, widely used in the assay of antidepressant drugs, was performed as described [83, 86]. The mouse was suspended in midair on a lab stand, by a strip of adhesive tape attached 1 cm from the end of its tail. The animal was observed for 6 min. Activity was recorded using a PC-based camera and the videos were later reviewed. Mice were considered immobile only when they hung passively and were completely motionless. The time spent motionless was the test endpoint. Data are presented with means ± standard deviations and statistical analysis was by a 2-way ANOVA, with genotype and sex the between-subject factors.

#### The forced swim test

The forced swim test, a well-validated assay for antidepressant drug action, was performed as described previously [82, 84–86]. A mouse was placed for 6 min in a 2-liter volume plastic cylinder, 21 cm in diameter, half-filled with water (22 °C), and observed for its mobility level. Activity was recorded using a PC-based camera and the videos were later reviewed. The time spent immobile (defined as floating in an upright position without any additional activity other than that necessary for breathing and keeping its head above water) was the test endpoint. Data are presented with means ± standard deviations and statistical analysis was by a 2-way ANOVA, with genotype and sex the between-subject factors.

#### Acoustic startle and prepulse inhibition

These assays were performed as described previously [126, 130]. Mice were placed into a clear plastic cylinder, left undisturbed for 5 min and then subjected to a 120 dB noise. Startle behavior was measured by a motion detector (Med Associates). For prepulse inhibition, the noise was preceded by a sound (i.e., the prepulse) 100 ms prior to the startle stimulus. The intensity (in dB above background) of the prepulse was progressively increased and its effect on startle response was measured. Testing consisted of 6 blocks of 7 trials each, with an inter-trial interval of 15 s. Data are presented with means ± standard deviations and statistical analysis was by a 2-way ANOVA, with genotype and sex the 2 between-subject factors.

#### Context-dependent and cue-dependent fear-conditioning

This was performed as described previously [126–128, 131–134]. It measured the ability of mice to learn to fear an emotionally-neutral conditioned stimulus (CS), such as a tone, temporally-associated with the aversive unconditioned stimulus (UCS), an electrical shock administered to the foot. For training, on day 1, each animal was placed in the fear conditioning chamber (Med Associates/Actimetrics chamber system) and allowed to explore for 3 min. A white noise (90 dB) “cue” was presented for 30 s, as the CS. During the last second of the cue, a mild foot shock (0.5 mA) was delivered for 1 s to the metal floor grid, as the UCS. Two pairings of white noise and foot shock were administered, separated by 90 s, after which the animal was returned to its home cage. On day 2, context-associated learning was assessed by recording freezing behavior during a 5-min exposure to the fear conditioning chamber (context test; Pre-CS) when no shock was administered. Cued fear conditioning (CS) was then tested by placing the animal in a different test cage with altered cage dimensions, colors, and smells. The animal was allowed to explore the new cage for 3 min, followed by presentation of the auditory cue for 3 min, and then freezing behavior was scored. Freezing behavior was recorded by a photobeam activity system (San Diego Instruments). Data are presented with means ± standard deviations and statistical analysis was by a 2-way ANOVA, with genotype and sex the 2 between-subject factors.

### Hippocampal slice electrophysiology

These studies were done using methods described previously by us [126, 127, 132, 134–139]. Transverse hippocampal slices (400 µM) from transgenic and control mice (8–12 weeks old) were prepared with a Vibratome. Electrophysiology was performed in an interface chamber (Fine Science Tools, Foster City CA, USA). Oxygenated (95%/5%::O_2_/CO_2_) artificial cerebrospinal fluid (ACSF) was perfused into the chamber at a rate of 1 ml/min. Slices were equilibrated in the chamber for 60–90 min at 30 °C prior to testing. Electrophysiological traces were digitized and stored using Digidata (models 1200 and 1320A) and Clampex software (Axon Instruments, Union City CA, USA). For measurement of LTP, extracellular stimuli were administered on the border of areas CA3 and CA1 along the Schaffer-collaterals using Teflon-coated, bipolar platinum electrodes. Field excitatory post-synaptic potentials (fEPSPs) were recorded in stratum radiatum of area CA3 with an ACSF-filled glass recording electrode (1–3 MΩ).

*Baseline stimulation* was done at 2 pulses/min, with each pulse lasting 0.1 ms. The relationship between fiber volley and fEPSP slopes over various stimulus intensities (25–1.5 nA) were used to assess baseline synaptic transmission. All subsequent experimental stimuli were then set to an intensity that evoked a fEPSP that had a slope of 50% of the maximum fEPSP slope. Responses (obtained at 0.05 Hz) were then monitored for at least 20 min, to ensure a stable baseline. *Paired*-*pulse facilitation (PPF)*, a measure of short-term synaptic plasticity [140] was performed as follows: Two stimuli were delivered to pre-synaptic cells, separated by a short interval. The stimuli were measured at various inter-stimulus intervals (20, 50, 100, 200, and 300 ms). PPF was calculated as the mean of the second response, divided by the mean of the first response. *Late*-*Long term potentiation* (L-LTP) was induced with 2 trains of 100 Hz tetani (each lasting 1 s), with an inter-train interval of 20 s between tetani. It has been shown that this treatment induces L-LTP in hippocampal slices from normal (wild-type) mice [126, 141]. Synaptic efficacy was monitored 0.5 h prior to and 3 h following induction of LTP by recording fEPSPs every 20 s (traces were averaged for every 2-min interval). Measurements are shown as the average slope of the fEPSP from 6 individual traces and standardized to the 20-minute baseline recording. *Statistics*: PPF and LTP were analyzed with 2-way ANOVA, with a P < 0.05. For each of these studies, 8–10 mice were tested in each group. The experimenter was blind to genotype in all behavioral experiments. All testing was done in the electrophysiology core at UAB.

## Abbreviations

αCaMKII: α-calmodulin kinase II
cAMP: 3′,5′ cyclic adenosine monophosphate
CREB: cAMP-response element binding protein
LDS-PAGE: lithium-dodecyl sulfate polyacrylamide gel electrophoresis
LTP: long-term potentiation
L-LTP: late long-term potentiation
PDE: 3′,5′ cyclic nucleotide phosphodiesterase
PDE4: 3′,5′ cAMP-specific phosphodiesterase 4
PKA: cAMP-dependent protein kinase
TBS: tris-buffered saline
TG: PDE4B1-D564A transgenic
VSV: vesicular stomatitis virus
WT: wild-type
UAB: University of Alabama at Birmingham
